# The evolution of an active solitary idiopathic choroiditis (focal scleral nodule): a case report of the natural course and a review of the literature

**DOI:** 10.1186/s12886-021-01888-5

**Published:** 2021-03-09

**Authors:** Yilin Feng, Christopher D. Conrady, Hakan Demirci

**Affiliations:** Department of Ophthalmology and Visual Sciences, Kellogg Eye Center, University of Michigan, 1000 Wall Street, Ann Arbor, MI 48105 USA

**Keywords:** Choroidal granuloma, Granuloma, Ocular mass, Solitary idiopathic choroiditis, Focal scleral nodule

## Abstract

**Background:**

To describe the clinical course of an active solitary idiopathic choroiditis (focal scleral nodule) that nearly resolved over six weeks without intervention.

**Case presentation:**

An 18-year-old man presented to the emergency department with headaches and new onset central scotoma in the right eye. Visual acuity was 20/20 in both eyes. Fundus examination revealed an amelanotic choroidal lesion with associated shallow subretinal fluid. It measured 6.1 × 6.3 × 1.4mm on A- and B-scan. Evaluation for systemic inflammatory and infectious diseases was negative. A week later, the lesion remained stable, and a month later, there was improvement of the lesion with a decrease in size on OCT and exam and resolution of the subretinal fluid suggesting that the lesion had become inactive.

**Conclusions:**

Solitary idiopathic choroiditis (Focal scleral nodule) is a rare condition characterized by inflammatory granulomatous reaction. This case report sheds light on the unknown natural course of a solitary idiopathic choroiditis (focal scleral nodule).

## Background

A focal scleral nodule (FSN), previously referred to as solitary idiopathic choroiditis (SIC) and unifocal helioid choroiditis, is characterized by an inflammatory granulomatous reaction in the sclera and/or choroid in the absence of an underlying systemic inflammatory disease [1–3]. It is a rare condition found more commonly in white females between the ages of 20 and 50 and where approximately a third of patients are asymptomatic at time of diagnosis [2].

Current management recommendations of FSN suggest monitoring of inactive lesions and either close observation or treatment with systemic corticosteroids for active lesions [2]. The natural history of an FSN has not been well documented, and to our knowledge, there are no specific case reports of FSN in the literature that describe the evolution of an active lesion to its inactive state. Here, we describe the clinical course of an active FSN that became inactive over six weeks without intervention.

## Case presentation

**Fig. 1:**
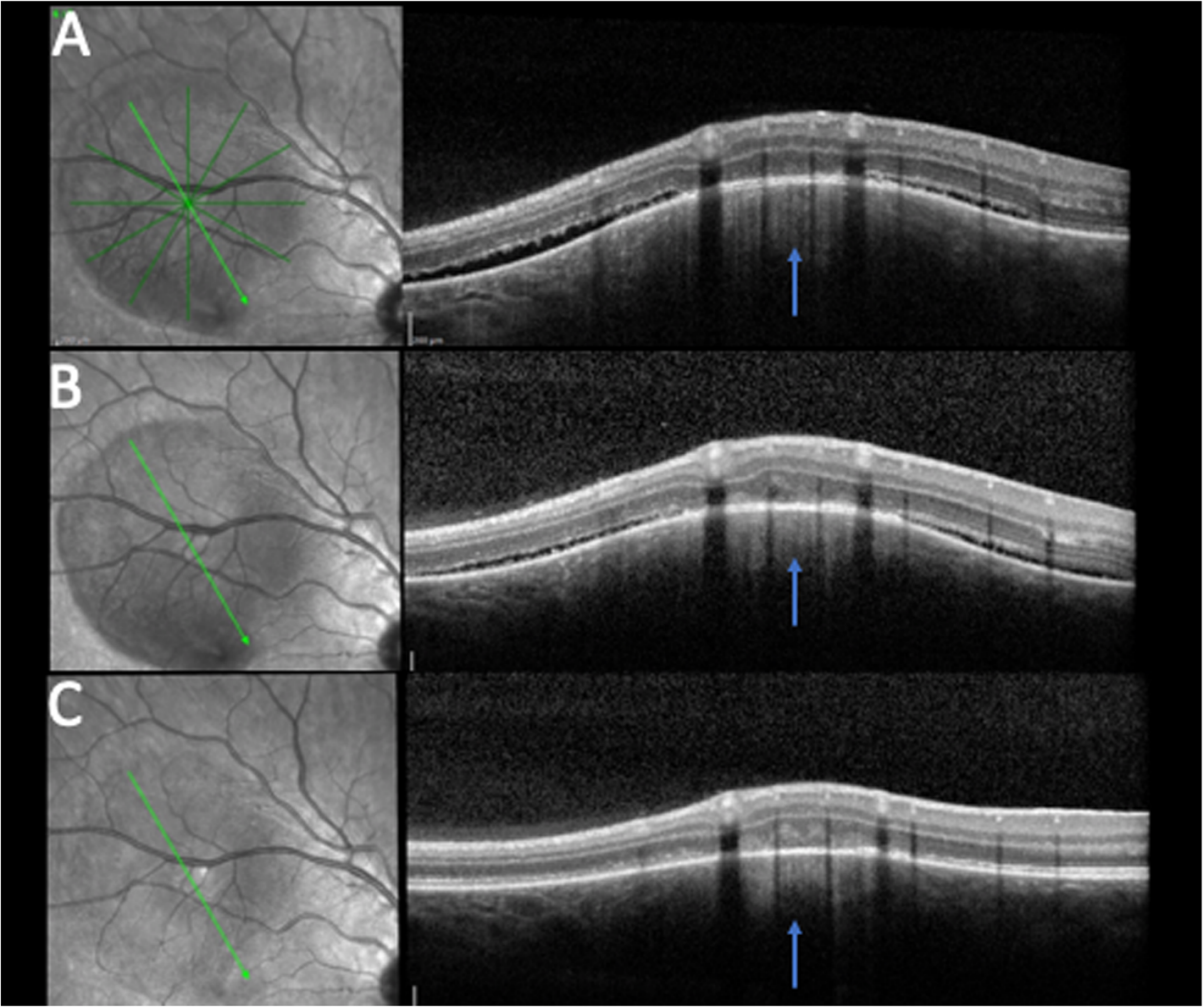
OCT findings at presentation, one week, and six weeks later. **a** OCT demonstrating SRF surrounding the elevated, choroidal lesion at presentation with a “contact sign” (retained attachment between retinal pigment epithelium and neurosensory retina over the granuloma) [4] and remained relatively unchanged one week later except for a mild improvement in the amount of subretinal fluid (**b**). **c** Six weeks later, the surrounding subretinal fluid had resolved and the choroidal lesion was much flatter. Blue arrow, lesion

**Fig. 2:**
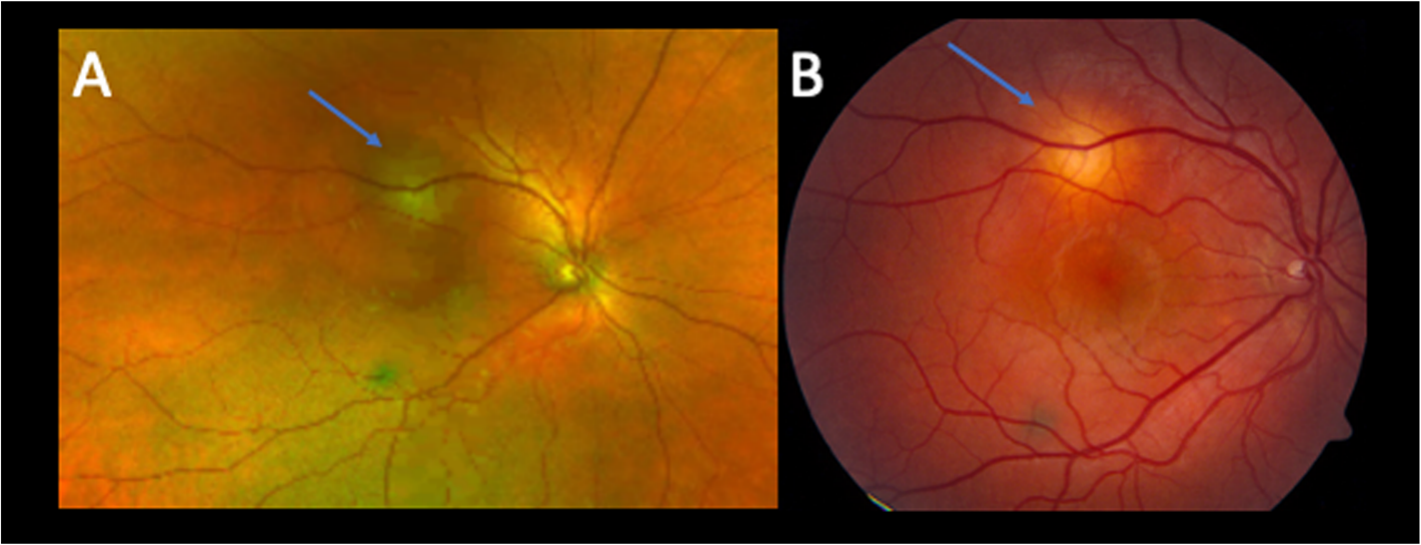
Fundus photographs at one week and six weeks later.**a** Fundus photograph showing a yellowish choroidal lesion with surrounding subretinal fluid one week after presentation. **b** Six weeks later, the subretinal fluid had resolved, and an orange halo had formed around the lesion. Blue arrow, lesion

**Fig. 3:**
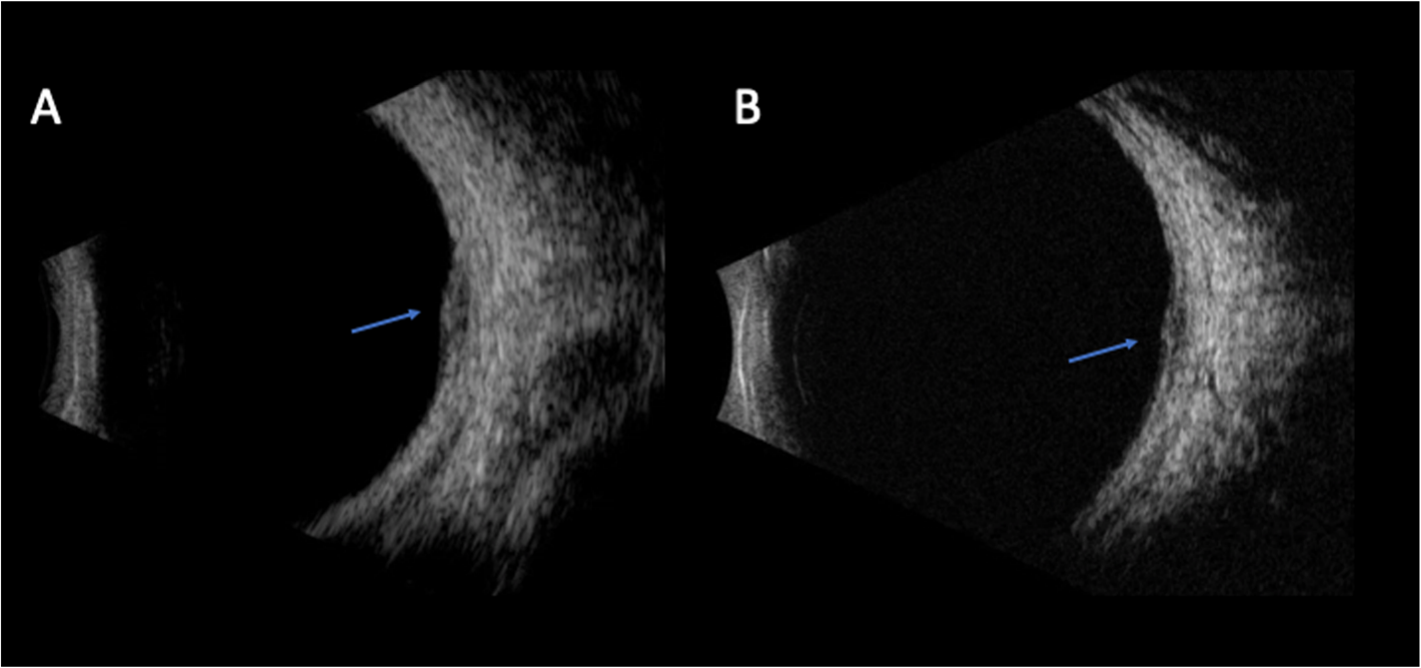
B-scan ultrasonography at one week and six weeks after presentation.**a-b** B-scan identified a small, dome-shaped fundus lesion measuring 1.4mm (height) x 6.1mm x 6.3mm one week after presentation (**a**) and remained relative unchanged six weeks later (**b**). Blue arrow, lesion

An 18-year-old otherwise healthy, white male presented to the emergency department with headaches and new onset central scotoma in the right eye of six days duration. He had a history of strabismus and amblyopia of the left eye as a child that had been surgically corrected twice. On initial presentation, his visual acuity was 20/20 in both eyes and anterior segment examination was unremarkable. Fundus exam at that time of the right eye demonstrated a round-shaped, amelanotic choroidal lesion, with associated shallow subretinal fluid (SRF) along the superior arcade. Optical coherence tomography (OCT) of the macula confirmed a hypointense choroidal mass with overlying outer retinal changes and SRF extending to the fovea (Fig. 1a). Due to the lack of systemic symptoms, a focused laboratory evaluation for infectious (syphilis, Lyme disease, Bartonella, toxocariasis, toxoplasmosis, and tuberculosis) and inflammatory diseases (sarcoidosis, and granulomatosis with polyangiitis) was unrevealing. The lesion was observed due to the patient’s good overall visual acuity and given the presumptive diagnosis of FSN. A week later and symptoms unchanged, diagnostic A- and B-scan ultrasonography demonstrated a dome-shaped acoustically-solid scleral/choroidal lesion with overlying SRF and intrinsic vascularity measuring 6.1 × 6.3 × 1.4mm, consistent with examination and OCT findings and stable from prior (Figs. 1b, 2a and 3a). Six weeks later, the symptoms of the patient had improved, the thickness of the lesion was relatively stable on A- and B-scan but improving on OCT, the SRF fluid had resolved, and an orange halo had formed around the lesion, suggesting that the lesion had become inactive (Figs. 1c, 2b and 3b).

## Discussion and conclusions

FSN is usually diagnosed based on detailed patient medical history, systemic laboratory evaluation for inflammatory and infectious etiologies, clinic features, such as lack of inflammatory cells in anterior chamber and vitreous, and ocular imaging findings such as absence of contact sign as reported in choroidal granulomas [4].

We herein describe a case of an 18-year-old, symptomatic male who presented with FSN. His FSN became inactive over the course of six weeks without intervention. Although this is a report of a single patient, we describe the natural course of an active FSN and its spontaneous resolution, which has not specifically been described in the literature. Due to the lack of formal guidelines on the treatment of FSN, the clinical course of patients who are followed longitudinally, as in our patient’s case, can provide valuable information to providers who encounter this rare condition.

In the largest retrospective study of FSN in the literature by *Shields et al.*., they reviewed sixty patients with both active and inactive lesions. In their study, the lesion remained stable in 60 % of the patients, improved in 37 %, and recurred in 3 % during a six-month to twenty-five-year follow up period (mean of 24 months) [2]. However, the results of this study were not stratified into active and inactive lesions, so the clinical course of an active lesion was still unclear. Furthermore, for the lesions that demonstrated improvement on follow up, the duration and course of improvement was also unclear due to the variable follow up length [2]. Moreover, 27 % of the patients in the study had received prior treatment, further confounding the study outcomes [2]. The authors of the study acknowledged the variation of patient treatments, stating that “it was impossible to determine the overall response to treatment [2].” However, they did suggest that active lesions generally respond favorably to systemic corticosteroids but can also improve without treatment. They also advised that inactive lesions should be monitored and that most remain quiescent over time [2].

Although there have been other reported cases of FSN, those reports also do not provide clarity regarding the natural course of an active lesion. Moreover, the authors frequently do not identify whether the presenting lesion is active or inactive. As seen in our case, the active lesion presents as a dull-yellow lesion with an ill-defined margin, associated with yellow intraretinal exudation, localized subretinal fluid, retinal vascular dilation, or focal retinal hemorrhages. In the original report of FSN by *Hong et al.*, a total of six patients were described, five of which received systemic corticosteroid therapy, and the sixth patient’s lesion was observed and remained stable after one year [1]. While this study originally described the entity, the use of systemic corticosteroids made it impossible to understand the natural history of the lesions [1]. In the report by *Kohne et al.*., they described a patient whose lesion mildly improved with no intervention six months later, but the lesion was likely inactive at presentation due to lack of surrounding fluid and distinct margins seen in the later phases of FA, features of activity [5]. Other case reports of FSN in the literature do not follow the patients longitudinally or present patients who have already received treatment making any extrapolation of natural history of the lesions impossible [6–8]. Understanding the evolution of active lesions can provide prognostic value and contribute to the recommendations of future treatment guidelines of FSN as we hope this case report does; however, as with any case report, this represents only a single case and broad generalizations cannot be drawn. Little is known about this entity, and recently, even the anatomical focus of inflammation has been re-defined [3].

## Data Availability

Data sharing is not applicable to this article as no datasets were generated or analyzed during the current study.

## Abbreviations

FA: Fluorescein angiogram
FSN: Focal scleral nodule
mm: Millimeter
OCT: Optical coherence tomography
SIC: Solitary idiopathic choroiditis
SRF: Subretinal fluid
